# Impact of Early Dressing Removal After Cesarean Section on Wound Healing and Complications: A Systematic Review

**DOI:** 10.7759/cureus.70494

**Published:** 2024-09-30

**Authors:** Zainab Al-Sulaitti, Bhavana Nelakuditi, Bindu Jyothi Dandamudi, Kathrina Antheia M Dimaano, Nensi Shah, Osamah AlQassab, Tuheen Sankar Nath

**Affiliations:** 1 Obstetrics and Gynecology, California Institute of Behavioral Neurosciences & Psychology, Fairfield, USA; 2 Internal Medicine, California Institute of Behavioral Neurosciences & Psychology, Fairfield, USA; 3 General Practice, California Institute of Behavioral Neurosciences & Psychology, Fairfield, USA; 4 Surgical Oncology, Tata Medical Center, Kolkata, IND

**Keywords:** cesarean section (c-section), duration of hospital stay, patient comfort, surgical site infections (ssi), wound care management, ­wound healing, wound infections

## Abstract

The global increase in cesarean section (C-section) births has heightened concerns about surgical site infections (SSIs), a significant risk associated with this common obstetric procedure. This literature review evaluates the impact of early dressing removal after C-sections, drawing from randomized controlled trials and clinical studies to assess potential benefits and risks. The review found no definitive evidence favoring a specific timing for dressing removal to reduce SSIs. However, earlier removal before hospital discharge generally enhances patient comfort. Factors such as high BMI, urgent C-sections, preterm premature rupture of membranes (PPROM), and chorioamnionitis are linked to higher wound healing complications, irrespective of when the dressing is removed. Removing staples before discharge is advised for convenience, with close monitoring recommended for high-risk patients. The findings emphasize the need for further research to optimize wound healing protocols, particularly for high-risk groups, to improve patient outcomes and minimize complications. A personalized approach to postoperative care, tailored to individual risk factors, may offer the best strategy for reducing SSIs and enhancing recovery after C-sections.

## Introduction and background

Cesarean section (C-section) is a surgical procedure used to deliver a baby through incisions made in the mother's abdomen and uterus. According to recent research from the World Health Organization (WHO), the global rate of C-section births continues to rise, currently accounting for over 21% of all deliveries. Projections indicate this trend will persist, with nearly 29% of all births expected to occur via C-section by 2030 [1]. As one of the most frequently performed obstetric procedures [2], C-sections carry significant intra-operative and post-operative risks, among which surgical site infections (SSIs) are particularly concerning. SSIs have been reported in 3-18% of patients following a C-section [3].

The Centers for Disease Control and Prevention (CDC) defines an SSI as an infection occurring at the site of surgery within 30 days post-procedure, or within one year if an implant is involved [4]. SSIs are among the most frequent healthcare-associated infections, occurring in 1% to 3% of all surgical procedures [5]. Their incidence is notably higher in abdominal surgeries, with rates ranging from 15% to 25%, depending on the level of contamination [5,6]. Although preventable, SSIs are associated with high morbidity and mortality, extended hospital stays, and increased healthcare costs [7,8].

Several factors can elevate the risk of SSIs, including the timing of dressing removal after surgery. However, evidence is insufficient to confirm whether covering surgical wounds reduces infection risk, and no definitive guidelines exist on the optimal timing for dressing removal [9]. Unnecessary and repeated dressing changes increase healthcare costs, burden nursing staff, cause patient discomfort, and disrupt wound healing [10,11]. Therefore, understanding the appropriate timing for dressing removal after C-sections is crucial for promoting optimal wound healing and reducing complications.

This literature review synthesizes current research findings on the impact of early dressing removal following C-sections. By examining randomized controlled trials and clinical studies, this review provides a comprehensive overview of the potential benefits and risks associated with early dressing removal, identifies gaps in the literature, and suggests areas for future research to inform clinical practices and improve patient outcomes.

## Review

Method

**Figure 1:**
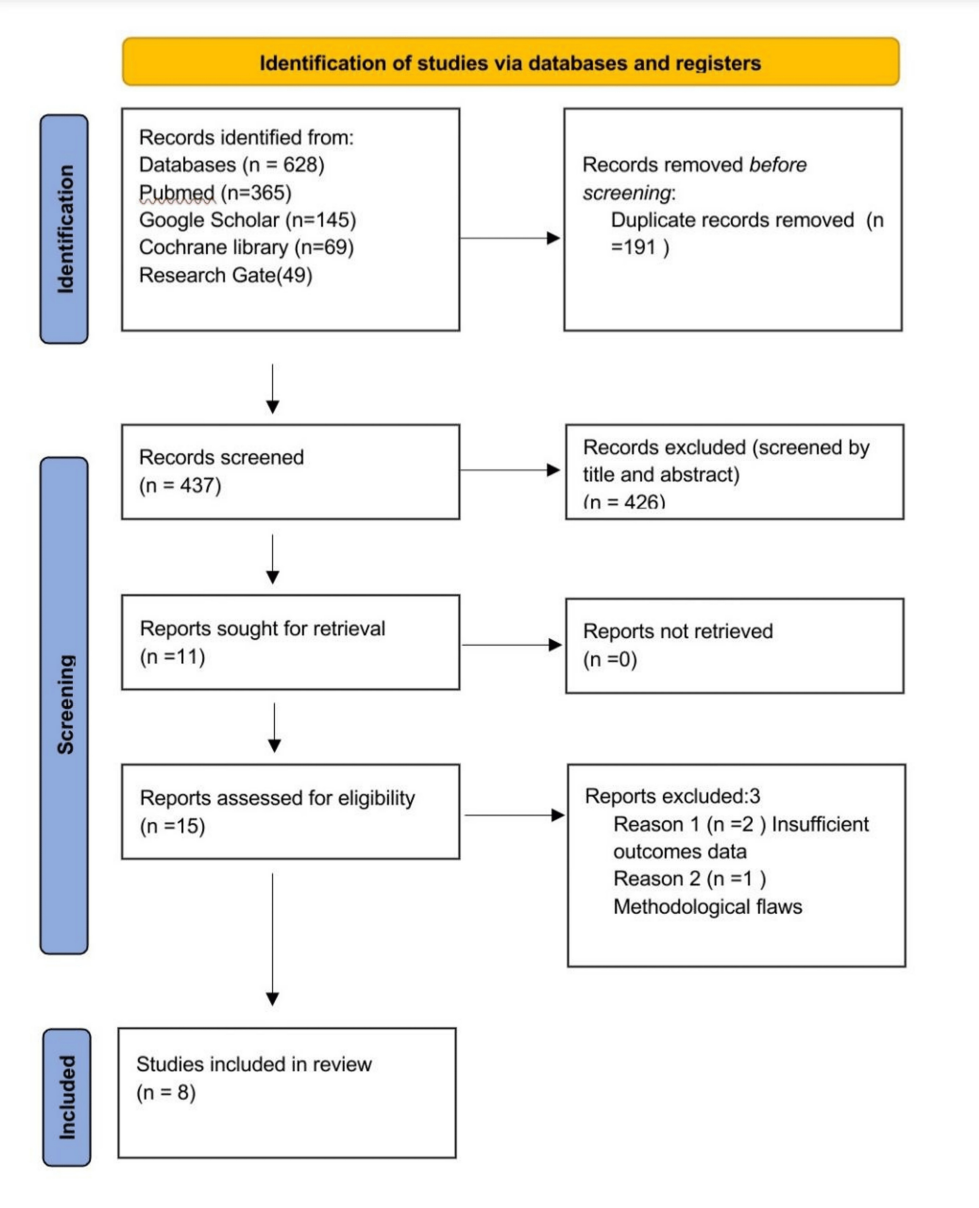
PRISMA flowchart PRISMA: Preferred Reporting Items for Systematic Reviews and Meta-Analyses

A comprehensive literature search was conducted across multiple databases, including PubMed, Google Scholar, Cochrane Library, and ResearchGate, to identify studies examining the impact of dressing removal timing after C-sections. The search focused on English-language publications from January 1, 2014, to June 24, 2024. A total of 628 citations were initially identified, and after removing duplicates, 437 unique articles remained. Title and abstract screening led to the exclusion of 426 studies, leaving 11 potentially relevant reports. Additionally, four publications from grey literature were retrieved for full-text review. After a thorough evaluation, three studies were excluded, resulting in eight studies that met the inclusion criteria. Data extraction was performed using a standardized form that captured the study design, sample size, outcomes, and key findings. Of the included studies, seven were randomized controlled trials (RCTs), and one was a clinical trial. The methods section is outlined according to the PRISMA (Preferred Reporting Items for Systematic Reviews and Meta-Analyses) 2020 flow chart, as depicted in Figure 1.

Search Strategy

A comprehensive literature search was conducted across PubMed, Google Scholar, Cochrane Library, and ResearchGate to identify studies on the impact of dressing removal timing after C-sections. The search strategy incorporated both controlled vocabulary and keywords, including ("Cesarean section" OR "C-section" OR "Cesarean delivery") AND ("early dressing removal" OR "early removal of dressing" OR "dressing removal timing") AND ("wound healing" OR "wound complications" OR "wound infection" OR "wound dehiscence" OR "postoperative complications"). The details of the search strategy are listed in Table 1.

**Table 1:** Strategy of the database search

Database	Search strategy
Pubmed	"Cesarean Section"[Mesh] AND "Cesarean Section, Repeat"[Mesh]) OR "Wound Healing"[Mesh]) AND ("Surgical Wound Infection/complications"[Majr] AND "Surgical Wound Infection/surgery"[Majr])) AND ("Surgical Wound Dehiscence/complications"[Mesh] OR "Surgical Wound Dehiscence/surgery"[Mesh])) AND ("Wound Infection"[Mesh] OR "Surgical Wound Infection"[Mesh])) OR "Time Factors"[Majr]
Cochrane	“Cesarean section AND early dressing removal”
Google Scholar	“Timing of dressing removal AND cesarean section”
Research Gate	“Early dressing removal AND wound complication after cesarean section”

Inclusion and Exclusion Criteria

Studies were selected based on stringent inclusion criteria, including publication in English, involvement of randomized controlled trials or clinical trials, focus on women aged 18 to 44, and inclusion of low-risk patients without comorbidities. Exclusion criteria encompassed high-risk populations, non-English articles, non-RCTs, and studies without full-text availability.

Primary and Secondary Outcomes

The primary outcome of interest was the influence of dressing removal within 24 hours or earlier on the rate of SSIs. Secondary outcomes included the impact of early dressing removal on patient comfort, mobility, and the duration of hospital stays.

The studies included in this traditional review consist of both RCTs and controlled trials (CTs), as outlined in Table 2, which details the characteristics of each study.

**Table 2:** Study characteristics

Authors and year of presentation	Intervention	Numbers of patients	Type of study	outcome	Conclusion
Kilic et al. (2020) [12]	The study compared the effects of removing wound dressings at 24 hours versus 48 hours after a cesarean section.	A total of 884 patients were enrolled and followed, with 869 completing the study. The 24-hour group included 430 patients, and the 48-hour group included 439 patients.	Multicenter, randomized controlled trial.	At six weeks post-surgery, the wound score was significantly lower in the 48-hour group (3.9%) compared to the 24-hour group (9%; p = .002). Patient complaints related to the incisions were significantly higher in the 24-hour group at six weeks (6.4%) compared to the 48-hour group (3%; p = .018).	Removing wound dressings at 48 hours post-cesarean section resulted in better wound healing and fewer patient complaints compared to removal at 24 hours. The study suggests that a 48-hour dressing removal protocol may be more beneficial for patients undergoing low-risk, scheduled cesarean deliveries.
Peleg et al. (2016)[13]	Early removal of wound dressing at 6 hours post-cesarean delivery vs. standard removal at 24 hours.	320 low-risk women aged 18-44 years.	Randomized controlled trial.	Incidence of wound complications: 13.8% in the 6-hour group vs. 12.5% in the 24-hour group (odds ratio, 1.16; 95% CI, 0.58-2.14). Patient satisfaction: 75.6% in the 6-hour group were pleased/satisfied compared to 56.9% in the 24-hour group (odds ratio, 2.35; 95% CI, 1.46-3.79).	Early removal of the wound dressing at 6 hours following cesarean delivery has no detrimental effect on wound healing. It allows women to attend to personal hygiene earlier, improving their satisfaction with postoperative recovery.
Tan et al. (2020) [14]	Comparison of exposed versus dressed transverse suprapubic cesarean wounds.	331 women delivered by cesarean section.	Randomized controlled trial.	Superficial SSI Rates: 1.3% in the exposed group (2/153) versus 3.2% in the dressed group (5/157) (relative risk [RR] 0.4, 95% CI 0.1–2.1; P = 0.45) Patient Satisfaction: Both groups reported similar satisfaction scores (7 [5–8], P = 0.81) · Patient Preference: Preference for wound exposure increased from 35.5% to 57.5% in the exposed group, while preference for a dressed wound decreased from 48.5% to 34.4% in the dressed group from recruitment to day 28.	The trial was underpowered as SSI rates were lower than expected. However, leaving cesarean wounds exposed does not appear to have detrimental effects, provided patient counseling is conducted to manage expectations.
Heinemann et al. (2019) [15]	The study investigates the timing of dressing and staples removal after cesarean delivery.	1394 women were recruited, and 920 completed the study.	Prospective clustered clinical trial.	The timing of dressing and staples removal did not significantly affect wound healing complications. Healing complications were more frequent in women with a BMI > 35 kg/m², urgent cesarean delivery, preterm premature rupture of the membranes (PPROM), and chorioamnionitis. Complication rates were similar across the clusters (18%-26%).	The timing of dressing and staples removal has no significant impact on cesarean delivery scar healing in both low- and high-risk parturients. However, high BMI, urgent CD, PPROM, and chorioamnionitis are associated with increased healing complications, irrespective of the timing protocols. Therefore, removing staples prior to discharge is recommended for convenience, and high-risk patients should be closely monitored postpartum. Further research is needed to improve wound healing in high-risk patients.
Wadhwa et al. (2021) [16]	Early Dressing Removal: Dressing was removed on the 4th day post-Cesarean section. Late Dressing Removal: Dressing was removed on the 8th day post-cesarean section.	Total Patients: 500 females. Early Dressing Removal Group: 250 patients. Late Dressing Removal Group: 250 patients.	Study Design: Prospective, randomized, controlled study.	Incidence of Superficial Surgical Site Infection (SSI): Early Removal Group: 16% incidence of SSI. Late Removal Group: 32% incidence of SSI. Statistical Significance: p-value < 0.001, indicating a significant reduction in SSI in the early removal group. Duration for Complete Wound Healing: Early Removal Group: 6.6 days. Late Removal Group: 10.6 days. Statistical Significance: p-value < 0.001, showing faster wound healing in the early removal group. Length of Postoperative Hospital Stay: Early Removal Group: 5.6 days. Late Removal Group: 10.08 days. Statistical Significance: p-value < 0.001, indicating shorter hospital stays in the early removal group.	Main Findings: Early removal of dressing after a cesarean section significantly reduces the incidence of superficial SSI, accelerates wound healing, and shortens the length of postoperative hospital stay. Overall Impact: The study suggests that early dressing removal improves patient outcomes by enhancing wound healing, reducing the risk of infection, and decreasing hospital stays, thereby improving the quality of life and reducing financial burden.
Khlifi et al. (2022) [17]	Group A: Dressing removed on the 2nd day postop vs. Group C: Dressing kept and changed every two days beyond 48 hours.	400 patients (200 in each group).	Prospective randomized study.	SSI rate: 3.5% in Group A vs. 10% in Group C (p=0.01) - Higher patient satisfaction in Group A: 94.5% vs. 70% (p<0.001) - Lower cost of infection management in Group A (p<0.001).	Maintaining dressing beyond 48 hours post-op is an independent risk factor for SSIs. Early removal of dressing on the 2nd day postop reduces infection risk, lowers costs, and increases patient satisfaction
El-Sayed et al. (2020) [18]	Early Exposure Group: Removal of wound dressing 6-12 hours after cesarean section. Delayed Exposure Group: Removal of wound dressing 5 days after cesarean section.	Total Participants: 128 women. Early Exposure Group: 64 women. Delayed Exposure Group: 64 women.	Randomized controlled trial.	Pain Levels: The early exposure group reported significantly lower pain levels compared to the delayed exposure group (P=0.008). Wound Complications: Slightly higher in the delayed exposure group, but not statistically significant (P>0.05). Comfort Levels: Higher in the early exposure group with significant differences (P<0.05). Hospital Stay: 90.6% of the early exposure group stayed in the hospital for one day, compared to 58.8% in the delayed exposure group, showing a significant reduction in hospital stay for the early exposure group (P<0.001). Wound Infection (SSI): No significant difference in SSI rates between the groups.	Early exposure of cesarean-section wounds reduces the incidence of wound complications and surgical site infections (SSI) with no significant difference compared to delayed exposure. It also significantly lowers pain levels and increases comfort among women. The study suggests that early wound dressing removal can be integrated as an evidence-based practice to reduce post-cesarean section wound infections and improve patient outcomes.
Chandrasiri et al. (2016) [19]	Intervention: Early removal of wound dressing between 6 to 12 hours after cesarean section. Control: Delayed wound exposure between 24 to 30 hours after cesarean section.	400 patients were randomized into two groups: 205 in the intervention group and 195 in the control group.	Stratified randomized controlled trial.	Surgical Site Infections (SSI): No significant difference in SSI rates between the early removal and delayed removal groups. Patient Comfort: Patients in the intervention group (early removal) were able to perform tasks such as sitting up, getting off the bed, walking, and squatting more easily compared to the control group (p < 0.001). Patient Acceptability: In the intervention group, 85% preferred early removal for future cesarean section, 78% felt it improved hygiene, and 90% believed it improved overall comfort.	Summary: Early removal of dressings from clean, primarily sutured cesarean section wounds (within 6 to 12 hours) does not increase the incidence of SSIs compared to removal within 24 to 36 hours. Patients with early exposure were more comfortable and could perform daily tasks more easily, and the majority found the early exposure method acceptable. The study supports the early exposure of cesarean section wounds but suggests larger multicenter studies to confirm these findings due to the small sample size of this pilot study.

Discussion

Updated CDC guidelines continue to recommend the removal of wound dressings between 24 and 48 hours after clean or clean-contaminated abdominal surgeries [20]. Recent literature has examined the surgical outcomes associated with various postoperative dressing removal timings, highlighting the critical role of this factor in influencing both primary and secondary outcomes in C-sections.

The timing of dressing removal after a C-section significantly affects primary outcomes, such as wound complications and SSIs, and secondary outcomes, including patient comfort, mobility, and hospital stay duration. This discussion will analyze the findings from several studies on early versus late dressing removal and explore the potential implications for clinical practice.

Wound Complications and SSIs

The primary focus of this analysis is the relationship between dressing removal timing and the incidence of SSIs, a crucial concern in postoperative recovery. Kilic et al. (2020) conducted a large multicenter randomized controlled trial (RCT) with 884 participants, comparing the removal of wound dressings at 24 hours versus 48 hours post-C-section [12]. The study revealed a significantly lower incidence of wound complications in the 48-hour group (3.9%) compared to the 24-hour group (9%; p = .002) [12]. This suggests that an additional day of coverage may offer better protection against infections during the initial stages of wound healing. The findings underscore the importance of not hastening dressing removal, particularly in controlled, low-risk elective C-sections where the risk of external contamination is minimized. However, patient-specific factors, such as overall health and surgical conditions, may necessitate an even longer dressing period.

Conversely, Peleg et al. (2016) investigated the outcomes of very early dressing removal at 6 hours post-surgery versus the standard 24-hour removal [13]. Their study, which included 320 low-risk women, reported an SSI incidence of 13.8% in the 6-hour group versus 12.5% in the 24-hour group (odds ratio, 1.16; 95% CI, 0.58-2.14) [13]. Although the difference was not statistically significant, the results indicate a slight increase in infection risk with earlier removal. This study highlights the potential risks of very early dressing removal, especially in settings with less stringent infection control. While early removal can enhance patient satisfaction and comfort, it must be balanced against the increased exposure of the wound to potential contaminants.

El-Sayed et al. (2020) further examined the impact of even shorter dressing removal times, comparing removal at 6-12 hours with delayed removal at 5 days [18]. Their RCT, involving 128 women, found no significant difference in SSI rates between the two groups, though a non-significant trend toward higher wound complications was observed in the delayed removal group [18]. This study challenges the conventional belief that early dressing removal heightens infection risk. However, the small sample size and lack of significant differences suggest that more extensive research is needed before these findings can be generalized. The study also raises the possibility that very early removal might be safe in specific settings, provided patients receive appropriate post-removal care and follow-up.

Khlifi et al. (2022) presented evidence against prolonged dressing retention, showing that early removal on the second day post-operation resulted in a significantly lower SSI rate (3.5%) compared to maintaining the dressing and changing it every two days beyond 48 hours (10%; p = 0.01) [17]. This suggests that extended dressing use may increase the risk of infection, potentially due to moisture accumulation and bacterial growth. The study strongly supports early dressing removal as a safe and effective strategy for reducing SSIs, particularly in resource-limited settings where prolonged hospital stays and frequent dressing changes may not be feasible.

In another study by Wadhwa et al. (2021), dressing removal on the 4th day post-C-section was compared with removal on the 8th day [16]. The study found a significantly lower incidence of SSIs in the early removal group (16% vs. 32%; p < 0.001) and faster wound healing [16]. This study provides strong evidence that early dressing removal can enhance wound healing and reduce infection risk, making it a critical intervention for improving patient outcomes and reducing healthcare burdens.

Finally, Heinemann et al. (2019) conducted a prospective clustered clinical trial examining the timing of dressing and staple removal [15]. The study found no significant difference in wound healing complications between different removal times but identified high-risk factors such as BMI > 35 kg/m², urgent C-sections, and chorioamnionitis as significantly increasing the likelihood of complications, regardless of the dressing protocol [15]. This study emphasizes the need for individualized care, particularly for high-risk patients. While timing may be less critical for low-risk individuals, those with additional risk factors require tailored approaches to minimize complications. This finding underscores the importance of flexible clinical protocols that consider patient-specific factors rather than a one-size-fits-all approach.

Patient Comfort, Mobility, and Hospital Stay

Beyond wound complications and SSIs, the timing of dressing removal also significantly impacts secondary outcomes such as patient comfort, mobility, and length of hospital stay. Peleg et al. (2016) found that earlier dressing removal (6 hours post-surgery) resulted in significantly higher patient satisfaction, with 75.6% of women expressing satisfaction compared to 56.9% in the 24-hour group (odds ratio, 2.35; 95% CI, 1.46-3.79) [13]. The ability to attend to personal hygiene earlier and regain a sense of normalcy likely contributed to this increased satisfaction. However, it is essential to ensure that the benefits of early removal are not overshadowed by an increased risk of wound complications, which could negatively impact long-term patient satisfaction.

Similarly, El-Sayed et al. (2020) found that early dressing removal (6-12 hours) significantly reduced pain levels and increased comfort scores compared to delayed removal (5 days). Additionally, the early removal group had a notably shorter hospital stay, with 90.6% of participants discharged after one day, compared to 58.8% in the delayed group (P<0.001) [18]. This study strongly supports the advantages of early dressing removal in enhancing patient comfort and reducing hospital stays. The reduced pain levels and increased comfort associated with early removal contribute to faster recovery and lower healthcare costs. However, careful monitoring is necessary to ensure that early discharge does not lead to increased readmission rates or delayed complications.

Chandrasiri et al. (2016) observed that early removal of dressings (6-12 hours) enhanced patient mobility, enabling them to perform activities such as sitting up, walking, and squatting more easily than those in the delayed removal group (24-30 hours) [19]. Improved mobility likely contributes to faster recovery and shorter hospital stays. This study highlights the importance of mobility in postoperative recovery, as enhanced mobility not only improves physical recovery but also helps prevent complications like deep vein thrombosis. However, the benefits of increased mobility must be balanced with the need for wound protection during the early stages of healing, particularly in patients with additional risk factors.

Kilic et al. (2020) reported that patient complaints related to incisions were higher in the 24-hour group at six weeks post-surgery (6.4%) compared to the 48-hour group (3%; p = .018) [12]. This finding suggests that while delaying dressing removal might initially limit mobility, it could reduce long-term discomfort. The study highlights the complexity of patient comfort, which is influenced by both short-term and long-term factors. While early removal might improve immediate comfort and mobility, a more conservative approach could be beneficial for reducing discomfort in the weeks following surgery. This finding suggests that the timing of dressing removal should be carefully considered based on both the patient’s immediate needs and their long-term recovery trajectory.

Wadhwa et al. (2020) further supported the benefits of early dressing removal, demonstrating that it not only reduces SSIs and accelerates wound healing but also significantly shortens hospital stays (5.6 days vs. 10.08 days; p < 0.001) [16]. The study provides compelling evidence for the advantages of early dressing removal, particularly in settings where reducing hospital stays and healthcare costs are priorities. The significant reduction in hospital stay times associated with early removal could lead to better resource allocation and improved patient turnover in busy healthcare settings. However, it is essential to ensure that these benefits are not outweighed by an increased risk of readmission or delayed complications, which could negate the advantages of shorter hospital stays.

Finally, Heinemann et al. (2019) emphasized that while the timing of dressing removal might not significantly impact wound healing for low-risk patients, it should be tailored to the needs of high-risk patients to optimize outcomes [15]. This study is a crucial reminder of the need for personalized care strategies. While early removal might be beneficial in many cases, high-risk patients require a more cautious approach to ensure that the benefits of early intervention do not come at the expense of increased complications.

Limitations

The studies reviewed included large cohorts, but some were underpowered or had limited sample sizes, potentially affecting the generalizability of their findings. For instance, Tan et al. (2020) conducted a study that was underpowered due to lower-than-expected SSI rates, indicating that the results may not fully capture the risks or benefits of early dressing removal [14]. Additionally, studies conducted within specific populations, such as low-risk women or particular geographic regions, may not be applicable to all patient groups. The heterogeneity of study designs, varying timing protocols, and diverse outcome measures introduce challenges in comparability. Differences in defining and measuring outcomes, such as SSIs, patient satisfaction, and mobility, contribute to inconsistencies in findings. Furthermore, while some studies, like Heinemann et al. (2019), accounted for patient-specific risk factors (e.g., high BMI, urgent C-section, preterm premature rupture of membranes), others did not, potentially underestimating risks for higher-risk populations [15]. The relatively short follow-up periods in certain studies may not fully capture long-term outcomes or late-onset complications associated with different dressing removal timings, suggesting that longer follow-up is necessary for a comprehensive understanding of these practices' impacts on wound healing and recovery. Lastly, while some studies were multicenter, others were conducted at single institutions, introducing potential bias related to institution-specific surgical practices or patient care protocols.

## Conclusions

The evidence suggests that earlier dressing removal, particularly within the first six to 24 hours post-cesarean section (C-section), does not increase the risk of surgical site infections (SSIs) and may offer substantial benefits in terms of patient comfort, mobility, and reduced hospital stay. However, slight delays in dressing removal, up to 48 hours, may provide additional protection against wound complications, as suggested by some studies. Given these findings, there is a clear need for the standardization of protocols regarding the timing of dressing removal to reduce variability in outcomes across diverse populations and healthcare settings. Further research should focus on high-risk populations, such as patients with obesity, diabetes, or those undergoing emergency C-sections, who may have different healing trajectories and could benefit from tailored dressing removal strategies. Additionally, longer follow-up studies are necessary to assess the long-term impact of early dressing removal on wound healing, patient satisfaction, and potential late-onset complications. To increase the robustness and generalizability of these findings, future studies should be multicenter trials involving diverse geographic regions and healthcare systems, facilitated by international collaboration. Healthcare providers should also integrate patient education and counseling about dressing removal timing into postoperative care to manage patient expectations and improve overall satisfaction with the recovery process. Balancing the risks and benefits, considering individual patient factors like BMI and the urgency of the C-section will be essential in refining these protocols to optimize patient outcomes.
